# Is there a better indicator for predicting the outcome of trial without catheter?

**Published:** 2007

**Authors:** R. Shanmugasundaram, Nitin S. Kekre

**Affiliations:** Department of Urology, Christian Medical College, Vellore - 632 004, Tamilnadu, India. E-mail: uro2@cmcvellore.ac.in

## SUMMARY

This prospective clinical study was done to assess the impact of intravesical prostatic protrusion (IPP) on the outcome of trial without catheter (TWOC) following acute urinary retention (AUR). Consecutive white men aged 50 years or older with AUR related to benign prostatic hyperplasia (BPH) were recruited for the study. The mean age of these men was 70.1 years. Men with neurological illness, prostatic carcinoma, abnormal renal function, urethral stricture, residual urine > 1500 ml, prostatic or urethral surgery, being treated with anticholinergics and with severe co-morbid illness were excluded. All were given 10 mg of Alfusozin daily after catheterization along with treatment of precipitating factors. TWOC was given after two weeks. The prostatic volume was measured with transrectal ultrasound of the prostate (TRUS) immediately after catheter removal. The mean prostatic volume (PV) of this cohort was large (68.3 ml) and their mean IPP was 12.4 mm. Fifty-seven out of 121 men with AUR fulfilled the selection criteria. Of these 57, 18 and 39 had precipitated and spontaneous AUR respectively. A strong correlation was noted between successful TWOC and prostatic volume and IPP. Twenty-five out of 57 (43.9%) had successful TWOC (8/18 in the precipitated group and 17/39 in the spontaneous group). Men with successful TWOC had smaller mean PV (55 *vs* 70 ml) and smaller mean IPP (7.2 mm *vs* 16.5 mm). No association was found between age, retention volume, mode of retention (spontaneous or precipitated) and successful TWOC. Men with PV ≥ 50 ml had a three fold risk of unsuccessful TWOC compared to those with PV < 50 ml (*P* < 0.002). From Receiver Operating Characteristics (ROC) curve analysis, IPP was more accurate than PV in predicting successful TWOC. Of the men with IPP ≤ 10 mm, 78% had successful TWOC compared with 13% success in those with IPP > 10 mm (P < 0.0001).

## COMMENTS

Trial without catheter after a short course of α-blockers is often adopted by many urologists for AUR. The predictors for a successful outcome following TWOC are residual urine < 500 ml, gland size < 50 ml, men younger than 65 years, TWOC after prolonged catheterization, lower urinary tract symptoms (LUTS) for less than six months and precipitated AUR.[1] Serum prostate specific antigen (PSA) as a surrogate marker for prostatic volume has also been found to predict the outcome of TWOC. The higher the PSA, the greater is the subsequent risk for unsuccessful TWOC and surgery.[2] In this prospective study on white men, the subjects had a larger mean prostatic volume of 68.3 ml. Men with successful TWOC had smaller prostates and smaller IPP. Whether IPP measurement alone, irrespective of the size of the prostate, can be used to predict the success of TWOC remains unanswered. However, an IPP > 10 mm even in smaller volumes of the prostate (average volume of 39 ml) has been shown to be a predictor of an unsuccessful outcome of TWOC by Tan and Foo in their Asian cohort.[3] IPP was measured by the transabdominal method and no alpha blockers were used in their study. Thus, an IPP < 10 mm has been proved to be a predictor of success after TWOC in both cohorts having people of various races. IPP was also proven to be correlating well with urodynamically proven bladder outflow obstruction. Men with a larger IPP and AUR are less likely to respond to alpha blockade and TWOC.[4] Irrespective of the method of estimation, an IPP > 10 mm in men presenting with AUR due to BPH should set off warning bells that they are unlikely to have successful TWOC and are to be counseled for prostatectomy. IPP may not only predict the success of TWOC outcome, but can also be used in prognostication and treatment of symptomatic BPH. The predicting ability of IPP in the success of TWOC needs to be further established by standardizing its method of estimation and correlating it with other variables like prostatic volume, PSA, residual urine and severity of LUTS in larger multicentric prospective studies.
